# Brazilian consensus in enuresis–recomendations for clinical practice

**DOI:** 10.1590/S1677-5538.IBJU.2019.0080

**Published:** 2019-01-29

**Authors:** José Murillo B. Netto, Atila Victal Rondon, George Rafael Martins de Lima, Miguel Zerati, Edison Daniel Schneider-Monteiro, Carlos Augusto F Molina, Adriano de Almeida Calado, Ubirajara Barroso

**Affiliations:** 1 Universidade Federal de Juiz de Fora Hospital e Maternidade Therezinha de Jesus Faculdade de Ciências Médicas e da Saúde de Juiz de Fora Juiz de Fora MG Brasil Universidade Federal de Juiz de Fora (UFJF) e Hospital e Maternidade Therezinha de Jesus da Faculdade de Ciências Médicas e da Saúde de Juiz de Fora (HMTJ-SUPREMA), Juiz de Fora, MG, Brasil;; 2 Universidade do Estado do Rio de Janeiro Hospital Federal Cardoso Fontes Rio de Janeiro RJ Brasil Universidade do Estado do Rio de Janeiro (UERJ) e Hospital Federal Cardoso Fontes (HFCF), Rio de Janeiro, RJ, Brasil;; 3 Hospital Infantil Albert Sabin Fortaleza CE Brasil Hospital Infantil Albert Sabin, Fortaleza, CE, Brasil;; 4 Instituto de Urologia e Nefrologia de São José do Rio Preto Faculdade Regional de Medicina Hospital de Base São José do Rio Preto SP Brasil Instituto de Urologia e Nefrologia de São José do Rio Preto (IUN) e Faculdade Regional de Medicina(FAMERP), Hospital de Base, São José do Rio Preto, SP, Brasil;; 5 Hospital Pontifícia Universidade Católica de Campinas Campinas SP Brasil Hospital da Pontifícia Universidade Católica de Campinas (PUC-Campinas), Campinas, SP, Brasil;; 6 Hospital das Clinicas Faculdade de Medicina de Ribeirão Preto Universidade de São Paulo Ribeirão Preto SP Brasil Hospital das Clinicas da Faculdade de Medicina de Ribeirão Preto da Universidade de São Paulo (HCFMRP-USP), Ribeirão Preto, SP, Brasil;; 7 Faculdade de Medicina Universidade de Pernambuco Recife PE Brasil Faculdade de Medicina da Universidade de Pernambuco (UPE), Recife, PE, Brasil ;; 8 Universidade Federal da Bahia Escola Bahiana de Medicina Salvador BA Brasil Universidade Federal da Bahia (UFBA) e Escola Bahiana de Medicina (BAHIANA), Salvador, BA, Brasil

**Keywords:** Enuresis, Urinary Incontinence, Lower Urinary Tract Symptoms

## Abstract

**Introduction:**

Enuresis, defined as an intermittent urinary incontinence that occurs during sleep, is a frequent condition, occurring in about 10% of children at 7 years of age. However, it is frequently neglected by the family and by the primary care provider, leaving many of those children without treatment. Despite of many studies in Enuresis and recent advances in scientific and technological knowledge there is still considerable heterogeneity in evaluation methods and therapeutic approaches.

**Materials and Methods:**

The board of Pediatric Urology of the Brazilian Society of Urology joined a group of experts and reviewed all important issues on Enuresis and elaborated a draft of the document. On September 2018 the panel met to review, discuss and write a consensus document.

**Results and Discussion:**

Enuresis is a multifactorial disease that can lead to a diversity of problems for the child and family. Children presenting with Enuresis require careful evaluation and treatment to avoid future psychological and behavioral problems. The panel addressed recommendations on up to date choice of diagnosis evaluation and therapies.

## INTRODUCTION

Enuresis is defined as an intermittent urinary incontinence that occurs during sleep, having clinical significance after the child completes 5 years of age (1). It is a frequent condition, occurring in about 15 to 20% of 5 years old children and 6.4 to 10.3% of children at 7 years of age (2-4) with a spontaneous resolution rate of about 15% per year (5) and will still be presented in approximately 0.5 to 2.3% of adults (6, 7).

Enuresis is a multifactorial condition. Hereditary factors have been described and genetic factors are the most important in the etiology of enuresis (8). Family history of enuresis plays important rule. Studies have shown a risk of a child have enuresis to be 44% if one parent was enuretic and 77% if both had enuresis, and 15% if neither one of the parents suffered from the disorder (9).

Other factors involved on enuresis physiopathology are changes in bladder function (nocturnal bladder overactivity) (10), nocturnal urinary output (nocturnal polyuria due to altered circadian cycle of the antidiuretic hormone) (11-13) and associated with disturbance of awakening (inability of the child to wake up in response to bladder contractions or full bladder sensation) (14, 15).

There is evidence that enuresis is associated with emotional and behavioral changes (16), dysfunctions of the urinary and intestinal tracts (17), and respiratory changes, such as nocturnal apnea (18-20).

About 20 to 30% of enuretic children present with psychological/psychiatric disorders (2 to 4 times more than non-enuretic ones) (21). Enuretic children present more behavioral problems than non-enuretic ones regarding to social and attention problems (22).

Many enuretic children will present emotional and/or behavioral changes, such as sadness, mood alteration, shame, low self-esteem, feelings of guilt, insecurity, social isolation, low school performance, among others (21). In addition, families are also affected and the consequences of enuresis, for both the child and his family, are often neglected. Recent studies have shown significant loss of quality of life, not only for children, but also for their families (23, 24). Thus, parents, by not well understanding the problem and also for being stressed often become intolerant and punishing the child. Two national studies have shown a high incidence of punishment in enuretic children, being it verbal, physical without contact (chastisement) or with contact (aggression) (25, 26).

When relevant emotional and behavioral changes are identified, especially in secondary enuresis, psychological evaluation and treatment are recommended.

**Figure 1:**
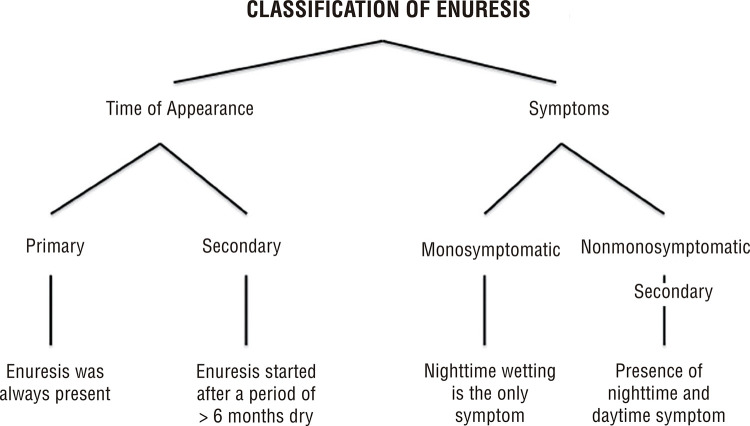
Classification of Enuresis.

Enuresis can be classified according to the moment of its appearance and to the symptoms presented and both classifications should be added to define the correct type of enuresis (Figure-1).

Evaluating the outcome of a treatment is important for clinical and research purpose. The standardization document from ICCS considers treatment outcome as “No Response when there is an improvement of less than 50% of the symptoms, “Partial Response” when there is improvement of 50 to 99% of the symptoms and “Complete Response” when 100% of the symptoms are resolved. Relapse is considered when more than one episode occurs per month. Continuous Success is when there is absence of relapse in 6 months and Complete Success when no relapses occur after 2 years (27).

## MATERIALS AND METHODS

The board of Pediatric Urology of the Brazilian Society of Urology, noticing the need of a Brazilian guideline on Enuresis, joined a group of experts to review the important issues on Enuresis and elaborated a consensus document. Eight renewed pediatric urologist with known experience in dealing with voiding dysfunction and enuresis were invited to participate in the elaboration a document with the scope of the guiding urologists, pediatricians, nephrologists and all others that deal with enuresis on the most important and up to date aspects of the evaluation and treatment of enuretic children.

All panel members were instructed to perform a literature search on MEDLINE, EMBASE and COCHRANE LIBRARY databases as well as review of the base of practical guidelines database for the last 20 years using the terms “Enuresis”, “Nocturnal Enuresis”, and “Bedwetting”. Papers were selected according to their level of evidence, giving more importance to meta-analysis, systematic reviews, and randomized controlled trials. Criterion of exclusion of bibliography included topics in which neither of those were found or were not of good quality or did not address treatment options. Cohort and series of patients were used to add information. Review papers and guidelines were used as orientation for which topics and aspects would be included.

After the papers were selected, each member of the group were designated one topic to review and write an orientation document based on the recommended literature.

On September 2018, all members joined together during 2 days to review and discuss the previous written documents of each topic and prepare the consensus document. Further discussions, corrections, and revisions were carried out digitally, until all members of the panel have approved this final document. A paragraph containing the panels opinion (“consensus”) was added at the end of each section to guide the reader about the information provided and the most common practice on that subject.

## CLINICAL EVALUATION AND DIAGNOSIS

A careful and meticulous clinical history is the best tool to understand and diagnose the correct type of enuresis and propose the most appropriate treatment. For this, it is necessary to differentiate between primary or secondary, and mono or non-monosymptomatic enuresis.

Anamnesis should include the child’s age, if any dry period had occurred, presence of voiding symptoms throughout the day (incontinence, increased voiding frequency, urinary urgency and low volume voiding), bowel habits, number of enuresis episodes per night and per week, information on child’s sleep pattern, sleep apnea, and difficulties in awakening, presence of any behavioral problem, such as attention deficit and hyperactive disorder (ADHD), anxiety, stress, abuse, bullying (28) and punishment (25). Past and familial histories are also important.

A careful history of bowel habits should be included to investigate constipation. The use of Rome IV criteria and Bristol Stool Scale is recommended to help making the proper diagnosis. In cases when the child presents signs of increase urine output diabetes mellitus should be investigated (glycosuria and glycaemia) and excluded.

Physical exam is helpful in identifying associated comorbidities. Genitalia should be careful examined and also abdomen, where signs of constipation (impacted feces in the left colon) can be found. Examination of the back is important to exclude any cutaneous sign of occult spinal cord malformation (29). As a complement to the clinical history, the child should be asked to complete both a voiding and a night diary. The voiding diary increases the reliability of the information given by the family and makes the parents aware of their child voiding habits. It should be performed for two to three days, not necessarily consecutive, and include all void and drink episodes. The voiding diary collects information on frequency, time of void, volume at each micturition, episodes of urgency and/or incontinence, liquid intake. It is considered normal 4 to 7 void per day, and an average of voided volume between 65 and 150% of the expected bladder capacity for the age, calculated by the formula ((Age+1) x 30) (27). A carefully orientation, explaining any doubts, on how to fulfill the voiding diary is important to avoid problems in its outcome.

A dry night diary of 14 consecutive days should also be obtained with the purpose of recording the frequency that enuresis occurs. To obtain the night-voided volume, the child is asked to sleep wearing a diaper. The sum of the diaper’s weight (Kg), the voided volume of the first micturition, and the voided volume of any episode of nocturia, if present will give the nocturnal diuresis. Night polyuria is considered to be a nocturnal diuresis volume >130% estimated bladder capacity or > the volume expressed by the formula ((age+9) x 20) (27).

No other test is necessary in the evaluation of monosymptomatic enuresis. Children with polyuria and polydipsia should be investigated for diabetes insipidus.

Evaluation of non-monosymptomatic enuresis will be discussed at the end of the document.

Consensus: The panel believes that a careful and meticulous clinical history considering all aspects discussed above, associated with a voiding diary are the most important tools in the evaluation of an enuretic child. It is important to address all behavioral problems the child may have. The addition of any other diagnostic test is rarely necessary in children with monosymptomatic enuresis.

## TREATMENT (Figure-2)

### Urotherapy

Urotherapy or behavioral therapy consists in a series of orientations indicated as initial treatment in all patients with enuresis it should be maintained throughout the treatment even when other therapeutic modalities are chosen (27, 30). The aims of urotherapy are to inform and demystify enuresis and give orientation on habits modifications to improve symptoms.

**Figure 2:**
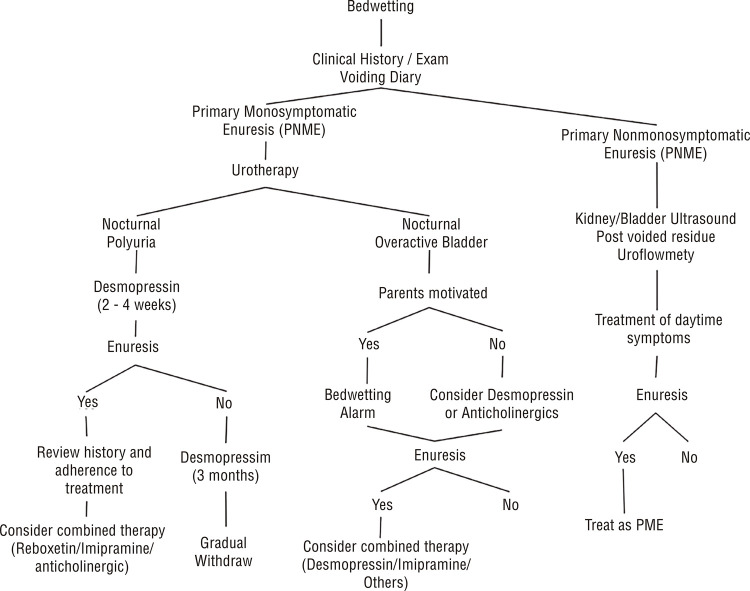
Treatment Algorithm.

Urotherapy orientation includes (27, 31): 1) information and demystification. Explanation about the normal function of the urinary tract and how that particular child deviates from the normal pattern; 2) regular voiding habits (voiding every 3 to 4 hours); 3) avoid retention maneuvers; 4) appropriate voiding position on the toilet to promote relaxation of the pelvic floor (appropriate-sized seat, footrest, forward bending of the torso); 5) regular bowel habits (standard time for evacuation, preferably after meals); 6) increase fluid intake during the day, specially in the mornings and early afternoon; 7) change dietary habits (eliminate caffeine, avoid citric fruits and juices, reduce sodium intake at nigh); 8) avoid any liquids at least 2 hours before bedtime; 9) void prior to go to bed; 10) no use of diapers; 11) dry nights calendar; and 12) support and encouragement through regular follow-up with the child and caregiver, providing positive reinforcement (award system for dry nights).

Behavioral interventions were superior when compared to no intervention, but had worse results when used alone to treat associated with alarm or drug therapy (32, 33). The resolution rate of enuresis with urotherapy alone is about 20% (34).

Consensus: The panel agrees that urotherapy orientations should be offered to any child with enuresis as first line treatment, regardless if it is mono or non-monosymptomatic. The health professional should provide clear information and reinforce all orientation given in every clinical consult. The addition of other therapeutic modalities will increase success of urotherapy.

### Enuresis Alarm

The Enuresis Alarm is an electronic device that uses a humidity sensor placed at underwear and connected to a sound and/or vibrating circuit that will be activated when the child wets the bed. The purpose of the alarm is to wake the child up during the enuretic episode and make the user (or a responsible person) go to the toilet to complete micturition. It is considered as behavioral or conditioning treatment. The age recommended to start treatment with alarm is six to seven years when the child is mature enough to accept and understand the treatment (35).

The alarm is considered the most effective long-term treatment for enuresis (Level of evidence and recommendation 1A). Results suggest a success rate of 62 to 75% after treatment, with a relapse rate of 15 to 30% during long-term follow-up (36-38).

The greatest problem with enuresis alarm is that a high number of patients discontinue treatment (30% to 50%) due to loss of motivation or other factors, such as waking up other members of the family during the night and the duration of the treatment that may take about six to eight weeks (38, 39). Due to this, a supportive approach to the family is necessary to reduce these numbers (35).

Results with alarm therapy improves if urine volume is greater than or equal to 65% of the expected capacity for age, in children who have more number of wet nights (40), and when parents and child are motivated (34). Factors related to failure of poor results are punitive parental reactions to the bedwetting (41), child behavioral problems, higher socioeconomic status (41), and if treatment is started out of the winter (41).

It is recommended that the child should keep using the alarm after 14 consecutive dry nights before stopping treatment.

Alarm should not be indicated when punishment is identified and should be used with caution in cases of night sweats in hot climate areas, due to activation of the alarm by humidity caused by sweat, and when the child sleeps with other children and a risk of bullying exists.

Compared to desmopressin, studies suggest that enuresis alarm is better in the long run, but some show that results may equivalent if the highest alarm dropout rate is considered (35, 39).

The addition of desmopressin to alarm treatment improves dry nights rate initially, but in the long term there is no benefit with this addition. Therefore, there is no recommendation that these methods be used together except in cases that fail when either one is used alone (42).

Treatment with alarm is better than oxybutynin alone and the addition of oxybutynin to the alarm does not improve the success rate in the initial treatment. This association is only indicated in refractory cases (43). Alarm was shown to be better than imipramine in improving the number of dry nights (44).

Regarding the two types of alarm, the body-worn alarm and the bed pad alarm, the alarm fixed on the body is preferred by the children (44). There is no evidence on which type of alarm is better than the other. Further studies need to be performed.

Consensus: The panel highly recommends the use of alarm in children with primary monosymptomatic enuresis that presents with a high number of wet nights and parents are motivated. A careful evaluation of the child and family, discarding behavioral problems and punishment is important for treatment success. Alarm treatment should be followed closely due to a high number of children that discontinue treatment. When no or poor response is found, other drugs such as desmopressin may be added to the treatment.

### Desmopressin

Desmopressin acetate is a synthetic analogue of the natural hormone, arginine vasopressin (antidiuretic hormone). It acts increasing the reabsorption of water through the renal tubules, leading to an increase in urinary osmolality and decreased diuresis (45, 46).

Desmopressin is a first-line treatment for enuresis with grade 1A of evidence. Its best results can be obtained in children with nocturnal polyuria (diuresis >130% estimated bladder capacity or > (age+9) x 20mL, adding the volume of the first void in the morning) (27) and normal bladder reservoir function (maximum voided volume greater than 70% of estimated bladder capacity for age) (47), and children with a greater age, and limited number of wet nights a week (31, 46).

Its administration is orally and should be taken one hour prior to bedtime and about 2 hours after dinner. It is recommended to start with a low dose of 0.1 to 0.2mg and adjust, when necessary, up to a maximum dose of 0.6mg/day, regardless of age or weight (48). One hour before taking the medication, children should stop fluid intake in order to avoid risk of hyponatremia and water intoxication, as well as obtain an optimal urine concentrating capacity (49).

Initial treatment should be maintained for 2 to 4 weeks in order to obtain maximum effect and if there is improvement in the number of dry night it should be continued for at least 3 more months. If the child is dry, withdrawn of the medication may be done gradually, which seems to reduce relapse (48, 49). In cases when symptoms worsen after beginning medication withdraw, dose should be increased again and treatment maintained for three more months.

The overall success rate of desmopressin is up to 65% and the relapse rate, especially if a gradual withdraw is not done, goes up to 80% (50). However, the outcome is lower in the long term follow-up.

Consensus: The panel believes that desmopressin should be used as first line treatment for all children with nocturnal polyuria and those that the use of enuresis alarm is not suitable. Dose should be increased gradually until dryness is achieved and treatment maintained for at least 3 months. There is evidence that withdrawn should be done gradually. Parents and child should be aware that, although rare, increase fluid intake and desmopressin may lead to side effects (hyponatremia and water intoxication).

## Other Drugs

### Anticholinergics

The use of anticholinergics in enuresis is limited (grade 1B evidence) (51, 52). Children with increased voiding frequency and low bladder volume (<65% of estimated bladder capacity) and with nocturnal bladder overactivity are the most suitable candidates as anticholinergics act inhibiting bladder contractions. The most commonly used anticholinergic in our setting is oxybutynin, although others such as solifenacine, and tolterodine can be used.

The results with anticholinergics in enuresis, although better than placebo, are poor when used as monotherapy and they should only be indicated if other treatments have failed (31). In these cases, anticholinergics can be helpful in up to 40% of the patients, especially in combination with desmopressin (53).

The major problem with the use of anticholinergics is, in addition to bad results, that a significant amount of patients present side effects, such as constipation, increased post-voided residual, dry mouth, among others. Therefore, when using anticholinergic drugs, constipation should first be assessed (53).

Consensus: In the panel’s opinion, anticholinergics should be used only in cases where no success with desmopressin and alarm was achieved and in selected patients with overactive bladder. Evaluation of constipation and post-voided residual should be done prior to prescription.

### Tricyclic Antidepressants

The leading tricyclic antidepressant prescribed for enuresis is imipramine. Randomized trials have shown that imipramine is slightly better than placebo, but due to safety concerns (cardiotoxicity) and side effects, it is considered to be a third line treatment for enuresis (Grade 1C evidence) (54-56), and should only be used in cases of failure of the first therapeutic options or when patients cannot afford them.

The mechanism of action of tricyclic antidepressants in enuresis is still controversial. It is known that they decrease the amount of time spent in REM sleep, stimulate vasopressin secretion, and relax the detrusor muscle.

Imipramine has shown to be more effective particularly for short-term outcomes, with fewer wet nights per week when compared to placebo (54, 57). Imipramine promotes a reduction of 1 wet night per week, with about 20% of children being dry for at least 14 consecutive nights. However, no sustained results are seen after ceasing treatment, and the majority of patients relapse after discontinuation of treatment (54).

Combination therapy including imipramine and anticholinergics showed improved results when compared to imipramine alone or placebo in the short and long term, with fewer relapses than imipramine monotherapy (54, 58). The combination of tricyclic antidepressants and desmopressin did not show any improvement in treatment results (54, 59).

It is advised to take the medicine 1h before bedtime, in the initial dosage of 10-25mg, and it can be increased in 25mg after 1 week of treatment. The maximum dose in children aged 6-12 years is 50mg/day and in those over 12 years of age up to 75mg/day. Every 3 months, treatment should be discontinued for at least 2 weeks to decrease the risk of drug tolerance and observation of therapeutic efficacy.

Imipramine presents limitations due to its cardiotoxicity with potential risk of cardiac conduction disorders and myocardial depression in overdose. Other possible side effects are possible behavioral changes, irritability and drowsiness, dizziness, headache, sweating, lethargy, sleep disturbance or restlessness, apathy, depression, among others.

Consensus: It is the panel’s opinion that imipramine and other tricyclic antidepressants should be reserved to those that failed other treatment. It should be used with caution and the family should be aware of possible side effects and the medication should be kept away from child’s reach. Due to its low cost compared to other treatment modalities, it can be an option in patients with low income. Combination with anticholinergics should be in mind to improve results.

### Alternative Treatments

So-called alternative, or rather unconventional, treatments such as acupuncture, hypnosis, homeopathy, herbalism, chiropractic, faradization, among others, have been tested but there is no scientific evidence to support their use.

The results with neuromodulation (transcutaneous electroneurostimulation) are controversial, and there may be a reduction in dry nights but no complete response in primary monosymptomatic enuresis. Further studies are need to validate its use in enuresis (60).

Consensus: The panelists agree that alternative treatment should not be used, except for electroneurostimulation, that could be tried in cases in which other therapies have failed.

## THERAPY RESISTANT ENURESIS

Some patients will not respond to alarm nor desmopressin and those are considered the therapy resistant ones, and, for them, other therapeutic modalities should be tried.

Assessment of these children includes a careful clinical history and physical exam trying to identify any missed information in their previous evaluation and treatment (49).

Possible factors related to poor therapeutic response should be careful investigated prior to considering the child resistant to treatment (61). Some questions should be addressed, such as: 1) is enuresis really monosymptomatic or daytime symptoms are present?; 2) does the patient present nocturnal polyuria?; 3) is the child reducing/stopping fluid intake at least 2 hours prior to bedtime; 4) is he/she following dietary orientation (low caffeine and citric intake)?; 5) is desmopressin being taking as prescribed (1 hour before bedtime and 2 hours after dinner)?; 6) is desmopressin dose adequate or it can be raised?; 7) is the alarm being used correctly?; 8) is there any behavioral disorder, such as attention deficit and hyperactivity disorders or others?

Children not responding to enuresis alarm correctly should be reinforced and reoriented on how the alarm works and how to be used and, those not motivated, other therapeutic options, such as desmopressin or other drugs should be started.

In desmopressin resistant patients, possible causes are excess urine solute due to increased sodium intake or altered sodium circadian rhythm or influence of vasoactive hormones and prostaglandins, such as renin, aldosterone and atrial natriuretic peptide. After defining the patient as resistant to desmopressin, we can divide them among those with absence of response despite decreased diuresis and those with nocturnal bladder overactivity as a possible etiology (61).

Therapeutic options for desmopressin resistant children includes indomethacin, a cyclooxygenase inhibitor, although significantly reduces nocturnal sodium, urea and osmotic excretion, no significant decrease in the number of wet nights was observed neither as mono therapy nor as combined to desmopressin (62, 63).

Furosemide has also been tried as a second line treatment for those resistant to desmopressin. It should be taken in the morning to increase diuresis and excretion of sodium and solutes during the day and, when associated to desmopressin, it has shown decrease of frequency of enuresis (64).

Reboxetine, an antidepressant with noradrenergic action, significantly reduced enuresis episodes in therapy resistant patients when compared to placebo, although must of the responses were partial (65).

Combination therapy is an option in those patients. The combined use of desmopressin and an anticholinergic agent is well tolerated and results in a significant improvement in enuresis episodes and the results suggest a better response if higher anticholinergic dose is used (up to 10mg) (66, 67). The effect of combined therapy of desmopressin and alarm is still controversial and immediate positive effect has been presented (68, 69), although some have lacked to show long term effect (42).

Consensus: It is the panel’s opinion that therapy resistant enuresis should always be addressed by an experienced professional in the field, since those cases requires expertise due to its complexity and poor results with the current available treatment options. A careful clinical history and physical exam are keys to achieve success in those cases.

## NON-MONOSYMPTOMATIC ENURESIS

Non-monosymptomatic nocturnal enuresis (NME) is characterized by the presence of diurnal symptoms associated with enuresis and is present in about 1/3 of all enuretic children (70). It has peculiarities that differ it from the monosymptomatic enuresis, as increased risk of urinary tract infection and greater association with constipation and emotional/behavioral disorders.

Those children with NME need proper evaluation and treatment. Greater attention should be taken on diurnal voiding symptoms (urgency, frequency, incontinence, straining to void) and constipation. Besides the voiding diary, evaluation of the post-void urinary residue by ultrasound and uroflowmetry are required to make the correct diagnosis and propose the ideal treatment. Assessment of bowel function with Rome IV criteria and Bristol Stool Scale should be careful evaluated as those children frequently present constipation.

Initial treatment should address daytime symptoms first focusing on treating the lower urinary tract dysfunction, constipation, and any behavioral disorder if present (1).

Urotherapy is the initial therapy and should be performed for at least one month. In the persistence of symptoms, more specific treatments are indicated and added to urotherapy. Therapeutic options include anticholinergics, alpha-blockers, electro neurostimulation, biofeedback, and botulin toxin.

Children who do not improve diurnal and nocturnal symptoms should continue with other therapeutic modalities that remain focused on the treatment of daytime symptoms such as increased dose of medication or combination of medications and of medication with other therapies (electro neurostimulation or biofeedback). Constipation, when present, should be assessed in the beginning of treatment. Improvement of bowel habits is a key point for improvement of voiding function.

If enuresis is still persistent after improvement of daytime symptoms; its treatment follows those described for monosymptomatic enuresis.

Consensus: The panel believes that children presenting with non-monosymptomatic enuresis should be evaluated as those presenting lower urinary tract dysfunction, which includes voiding diary, uroflowmetry and ultrasound with evaluation of post-voided residual. Assessment of constipation and behavioral disorders should not be forgotten during clinical investigation and should be treated if present. Daytime symptoms are the focus of the initial therapy and enuresis is treated after improvement of daytime lower urinary tract symptoms.
